# Long term outcome of anastomotic leakage in patients undergoing low anterior resection for rectal cancer

**DOI:** 10.1186/s12885-020-07109-4

**Published:** 2020-08-20

**Authors:** Alice Artus, Nicolas Tabchouri, Othman Iskander, Nicolas Michot, Olivier Muller, Urs Giger-Pabst, Pascal Bourlier, Céline Bourbao-Tournois, Aurore Kraemer-Bucur, Thierry Lecomte, Ephrem Salamé, Mehdi Ouaissi

**Affiliations:** 1 Department of Digestive, Oncological, Endocrine, Hepato-Biliary, Pancreatic and Liver Transplant Surgery, Trousseau Hospital, Chambray les Tours, Avenue de la République, Chambray les Tours, France; 2 Department of General-, Visceral- and Transplant Surgery, University of Münster, Münster, Germany; 3 Department of Hepatogastroenterology and Digestive Oncology, Tours, France

**Keywords:** Rectal cancer surgery, Anastomotic leakage, Local recurrence, Low anterior resection score, Functional outcome, Long-term outcome

## Abstract

**Background:**

The influence of anastomotic leakage (AL) on local recurrence rates and survival in rectal cancer remains controversial. The aim of this study was to analyze the effect of asymptomatic anastomotic leakage (AAL) and symptomatic anastomotic leakage (SAL) on short- and long-term outcome after curative rectal cancer resection.

**Methods:**

All patients who underwent surgical resection of non-metastatic rectal cancer with curative intent from January 2005 to December 2017 were retrospectively analyzed. Short-term morbidity, long-term functional and oncological outcomes were compared between patients with SAL, AAL and without AL (WAL).

**Results:**

Overall, 200 patients were included and AL was observed in 39 (19.5%) patients (10 AAL and 29 SAL) with a median follow-up of 38.5 months. Rectal cancer location and preoperative neoadjuvant treatment was similar between the three groups. Postoperative 30-day mortality rate was nil. The permanent stoma rate was higher in patients with SAL or AAL compared to WAL patients (44.8 and 30% vs 9.3%, *p* < 0.001). The mean wexner continence grading scale was significantly different between AAL (11,4 ± 3,8), SAL (10,3 ± 0,6) and WAL (6,4 ± 4,7) groups (*p* = 0.049). The 3 and 5-year overall and disease-free survival rates were similar between the 3 groups (86.6% /84% vs 100%/100% vs 76%/70 and 82.9%/77% vs 100%/100% vs 94.7%/88.3% for patients with SAL, AAL, and WAL, *p* = 0.480 and *p* = 0.527).

**Conclusion:**

The permanent stoma rate was significant higher in patients with SAL or AAL compared to WAL patients. AL did not impair long-term oncological outcome.

## Background

The management of rectal cancer has substantially improved and standardized in the past twenty years. Preoperative chemoradiotherapy (CRT) followed by total mesorectal excision (TME) is now the gold standard for locally advanced rectal carcinoma [1–4]. Indeed, TME has led to decreased local recurrence rates and increased cancer-specific survival rates [5]. More recently, technical advances include also an intersphincteric approach for resection [6, 7] which, combined with a neoadjuvant treatment [4], aims to reduce the permanent stoma rate in patients undergoing low rectal cancer surgery. Despite numerous improvements, anastomotic leakage (AL) remains the most dreaded complication following rectal resection [8–10]. Its occurrence is associated with impaired postoperative functional results (anal incontinence, low anterior resection syndrome, [11] and ultimately an increased rate of permanent stomata (reaching nearly 30%)) [12–14]. Furthermore, AL is also associated with impaired oncological results with increased local and/or distal recurrence rates as well as decreased overall survival rates [15–17]. However, it is important to discriminate symptomatic from asymptomatic AL since these two conditions do not seem to have the same oncological and functional postoperative outcome [16]. Although several previous studies did not find a difference between without (AL) and asymptomatic anastomotic leakage (AAL) with regard to early postoperative functional and oncological outcome, these studies did not analyze and report long-term data from patients with AAL. Furthermore, these previous studies analyzed heterogeneous patient populations including also patients suffering from distant metastasis [11, 12, 18]. It is obvious that the inclusion of such patients has a negative impact on anastomotic healing rates, increases the risk for a permanent stoma and reduced recurrence-free and overall survival rates, respectively [14]. Therefore, the objective of our study is to report and analyse long-term oncological and functional outcome in a homogeneous population of patients with AAL and SAL who had undergone curative surgical resection for non-distant metastatic rectal cancer in a tertiary referral centre.

## Methods

### Study population

Retrospective study at a single tertiary referral center of patients who underwent curative intended surgery for adenocarcinoma of the rectum (upper rectum (10 to 15 cm); mid (5 to 10 cm); low (2 to 5 cm) from the anal verge) between January 2005 to December 2017. Patients suffering from distant metastatic disease or local tumor extension requiring resection of adjacent organs (genito-urinary or vascular structures) were excluded. Further exclusion criterion was underlying inflammatory bowel disease, familial adenomatous polyposis, or pelvic recurrence after primary surgery. Data were collected from medical records for each patient and included demographic parameters, primary tumor characteristics, neoadjuvant treatment, intraoperative and pathology variables, as well as short-term and long-term oncological and functional outcomes.

### Neoadjuvant treatment

Rectal cancer staging included digital rectal examination, recto-sigmoidoscopy, total colonoscopy, endorectal ultrasound and/or pelvic resonance imaging (MRI). All patients had thoraco-abdominal computed tomography (CT-scan). The upper (L5/S1 vertebrae) and the lower (levator ani/anal verge) tumor limit was assessed by MRI and/or endorectal ultrasound, respectively. Neoadjuvant therapy consisted of either long-course treatment or short-course radiotherapy. In case of combined radio-chemotherapy (CRT), patients received 50 Gray (Gy) radio-therapy given in 25 fractions over five weeks with concomitant chemotherapy using Capecitabine (Xeloda®). Surgery was scheduled eight to 10 weeks after the end of combined radio-chemotherapy [19]. Short-course radiotherapy included 25 Gy delivered in five fractions over a time span of five to seven days [20] followed by curative surgery one week later [21].

### Surgical procedures

All patients had preoperative mechanical bowel preparation [22]. Laparoscopic tumor resection was the standard approach. However, patients with T4 tumors underwent primary open tumor resection. A medial-to-lateral approach was the technique of choice. The inferior mesenteric vein was ligated at the inferior border of the pancreas, followed by the mobilization of the left colon, the splenic flexure (the extend of mobilization was left at discretion of the surgeon) and ligation of the inferior mesenteric artery. If possible, the left colic artery was preserved. The rectum was transected by the use of liner stapler in double stapling technique. The proximal colon was transected approximately 10 cm above the upper border of the tumor. The specimen was retrieved from the abdominal cavity via a small abdominal incision [23]. Mechanical colorectal or manual colo-anal anastomoses (side-to-end or end-to-end) were performed depending on tumor level. Patients with rectal cancer of the upper rectum underwent partial mesorectal excision with a distal 5 cm margin from the lower border of the tumor. All other patients underwent standard TME. In case of very low rectal cancer, a total or partial intersphincteric resection was performed when ever feasible [7]. A closed suction drainage was placed in the small pelvis for 48 to 72 h postoperatively. A protective stoma (ileostomy) was performed routinely. In selected patients with upper rectal cancer with no neoadjuvant radio-chemotherapy, and the intraoperative conditions were assessed by the surgeon as favorable, no deviation ileostomy was placed.

### Short-term outcomes

Any postoperative clinical (sepsis, peritonitis, emission of gas, pus, or feces from the pelvic drain, purulent discharge per anus, or rectovaginal fistula) and/or biological suspicion (increased CRP and white blood cells count) of AL led to an earlier CT-scan assessment. In case of asymptomatic AL, antibiotic treatment was given to all patients. If required, CT-scan guided, trans-anal drainage placement or even redo-surgery was performed. After hospital discharge, all patients underwent structured outpatient follow-up including physical examination, routine blood tests (including CRP) and a CT-scan with water-soluble contrast enema within three months postoperatively. Anastomotic leakage was defined and graded according to the International Study Group of Rectal Cancer [24]. Patients were divided in three groups as follows: symptomatic AL (SAL, including grade B and C ALs), asymptomatic radiologic AL (AAL, diagnosed on enema-contrast CT-scan, routinely performed 6 to 8 weeks postoperatively, before considering stoma reversal) and without AL (WAL). Ileostomy closure was scheduled six to eight weeks postoperatively if a control CT-scan with water soluble contrast enema showed no AL. In patients with AAL, stoma reversal was delayed until CT-scanning results showed no signs for AL or after a time interval of at least 6 months following rectal resection, as described recently [18]. Short-term 30-day postoperative complications were graded according to the Dindo-Clavien Classification [25]. In-hospital stay was measured from the day of surgery including the day of hospital discharge.

### Pathological results

Surgical specimens were analyzed using a standardized protocol [26]. Tumors were staged using the TNM classification according to the 8th edition of American Joint Committee of Cancer (AJCC). Circumferential and distal margins were defined as positive (R+) when 1 mm or less (primary tumor nodes or tumor deposit within the mesorectum) and as negative (R0) when greater than 1 mm. Total or partial mesorectal excision, colloid component degree, differentiation grade as well as presence of vascular, lymphatic or peri nervous emboli were stated in pathology report.

### Long-term outcomes

Long-term functional and oncological outcomes were recorded in all patients. Patient follow-up was performed every 3 months for the first 2 postoperative years, every 6 months for the next 3 years and then annually thereafter. Follow-up was updated until December 2018 and consisted of clinical examination, CT-scan, and blood tests with colonoscopy performed one and then every 3 years after surgery. Local recurrence was defined as tumor recurrence at the anastomotic site or in the pelvic cavity, while distant recurrence was defined as tumor recurrence beyond the loco-regional area including liver, lung and/or other extra pelvic sites. Follow-up data was obtained from medical records, outpatient clinic or phone interview. Long-term functional genito-urinary and digestive outcomes as well as quality of life assessments were recorded using a French translation of the low anterior resection score [27], the Wexner continence grading scale [28], the IIEF-5 erectile dysfunction score [29], the SF36 health survey [30], a French validated version of the European Organization for Research and Treatment of Quality of Life Questionnaire for Colorectal Cancer (EORTC QLQ-CR30) [31], the EUROQOL non disease-specific instrument for evaluation health-related quality of life [32] and the Fecal Incontinence Quality-of-life (FIQL) scale [33]. All previously mentioned surveys and questionnaires were collected in outpatient clinic or by phone interview whenever possible. For all functional scales, a greater score represented a more impaired quality of life and for all symptom scales, a greater score was associated with a greater severity of symptoms. Furthermore, surgery-related long-term complications such as small bowel occlusion, anastomosis stenosis and incisional hernias were also recorded. Patients who were unable to undergo protective stoma reversal and those who required secondary stoma placement (colostomy or ileostomy) after protective stoma reversal were also identified. Pathological response to neoadjuvant chemotherapy was considered partial if any tumor downstaging was noted on specimen, total if no residual tumor was found and absent if no downstaging was noted.

### Statistical analysis

Baseline and demographic characteristics of the studied population, intraoperative and pathological characteristics as well as short- and long-term postoperative outcomes were analyzed. Patients who presented with SAL, AAL and without AL (WAL) were compared. Categorical variables were compared using the χ2 test with Bonferroni correction whenever necessary. The Kaplan-Meier method was used to estimate recurrence-free survival (RFS) and overall survival (OS), which were compared using the Log-rank test. All statistical analyses were performed using SPSS version 20.0 (SPSS Inc., Chicago, IL) and statistical significance was accepted at the 0.05 level. Continuous variables were compared using ANOVA or nonparametric ANOVA tests, accordingly. Significant prognostic factors associated with permanent stoma were identified by univariable logistic regression and included in a multivariable analysis to determine independent risk factors. This study was conducted according to the ethical standards of the Committee on Human Experimentation of our institution and reported according to the Strengthening the Reporting of Observational Studies in Epidemiology (STROBE) guidelines [34].

## Results

### Patients’ characteristics

During the study period, 747 patients underwent surgery for rectal cancer in our department. Among these patients, 247 (33%) presented with synchronous metastasis (i.e. liver, pulmonary, peritoneal and/or other organs) and 300 (40%) underwent abdominoperineal resection and/or combined organ resection, and were therefore excluded. A total of 200 patients who underwent sphincter-preserving resection with curative intent for upper (53 patients (26.5%)), mid (105 patients (52.5%)) and low (42 patients (21.0%)) rectal adenocarcinoma, were included. Patients’ baseline characteristics are reported in Table 1. Overall, there were 137 (67.5%) men with a median age of 67 (IQR 59–73) years. Patients’ comorbidities were comparable between the three groups (ASA score, *p* = 0.306). Neoadjuvant therapy was administered in 150 (75.0%) patients, of whom 138 (92.0%) underwent long-course radiation therapy and 12 (8.0%) short-course radiation therapy, with no differences found between the SAL, AAL and WAL group (*p* = 0.855). Overall, 188 (94.0%) patients underwent laparoscopic procedure of whom 45 (22.5%) required conversion with no differences noted between the three groups (*p* = 0.707 vs *p* = 0.827). Total mesorectal excision was performed in 149 (74.5%) patients with no differences found between the three groups (*p* = 0.438). In patients sufering from low rectal adenocarcinoma, 9/42 patients underwent intersphinteric resection (one total and 8 partial) and 29/42 patients had a manual coloanal anastomosis with no differences found between the three groups (*p* = 0.885 vs *p* = 0.296). A diverting stoma was placed in 179 (89.5%) patients with no differences found between the three groups (*p* = 0.735). Detailed intraoperative variables are reported in Table 2.

**Table 1:** Demographic and perioperative characteristics

N (%)	Overall population	Symptomatic AL (SAL)	Asymptomatic AL (AAL)	Without AL (WAL)	P
	200 (100)	29 (14.5)	10 (5.0)	161 (80.5)	
Age (years). median ± IQR	67 (59–73)	67 (58–73)	70 (67–78)	66 (59–73)	
≥ 60 years. n (%)	145 (72.5)	21 (72.4)	8 (80.0)	116 (72.0)	0.526
< 60 years. n (%)	55 (27.5)	8 (27.6)	2 (20.0)	45 (28.0)	
Sex ratio (Female/Male)	65/135	7/22	3/7	55/106	0.561
BMI (kg/m2). median ± IQR	24 (23–28)	24 (23–26)	30 (25–34)	24 (23–27)	**0.017**
ASA Score. n (%)					
1	107 (53.5)	16 (55.2)	2 (20.0)	89 (55.3)	
2	79 (39.5)	11 (37.9)	7 (70.0)	61 (37.9)	0.306
3	14 (7.0)	2 (6.9)	1 (10.0)	11 (6.8)	
Arteriopathy. n (%)	16 (8.0)	2 (6.9)	2 (20.0)	12 (7.5)	0.355
Diabetes. n (%)	23 (11.4)	4 (13.8)	1 (10.0)	18 (11.2)	0.862
Tumor diagnosis. n (%)					
Screening test	41 (20,5)	8 (27.6)	1 (10.0)	32 (19.9)	0.447
Symptoms	159 (79.5)	21 (72.4)	9 (90.0)	129 (80.1)	
Rectal adenocarcinoma location, n (%)					
Upper (10–15 cm)	53 (26.5)	5 (17.2)	1 (10.0)	47 (29.2)	
Mid (5–10 cm)	105 (52.5)	18 (62.1)	8 (80.0)	79 (49.1)	0.308
Low (2–5 cm)	42 (21.0)	6 (20.7)	1 (10.0)	35 (21.7)	
Neoadjuvant radiation therapy, n (%)					
Long-course radiotherapy (with chemotherapy)	138 (69,0)	19 (65.5)	8 (80.0)	111 (68.9)	
Short-course radiotherapy (without chemotherapy)	12 (6.0)	2 (6.9)	0	10 (6.2)	0.855
None	50 (25.0)	8 (27.6)	2 (20.0)	40 (24.8)	

*AL* Anastomotic leak, *SAL* symptomatic AL, *AAL* asymptomatic AL and *WAL* without AL; *ASA* American Society of Anesthesiologists, *BMI* body mass index, *IQR* interquartile range

**Table 2:** Operative details

N (%)	Overall Population	Symptomatic AL (SAL)	Asymptomatic AL (AAL)	Without AL AL)	P
	200 (100)	29 (14.5)	10 (5.0)	161 (80.5)	
Laparoscopic, n (%)	188 (94.0)	27 (93.1)	10 (100.0)	151 (93.8)	0.707
Conversion to laparotomy	45 (22.5)	6 (20.7)	3 (30.0)	36 (22.3)	0.827
Complete TME, n (%)	149 (74.5)	22 (75.9)	8 (80.0)	119 (73.9)	0.438
PME, n (%)	51 (25.5)	7 (24.1)	2 (20.0)	42 (26.1)	
Rectal anastomosis technique, n (%)					0.310
Latero - terminal	93 (46.5)	13 (44.8)	7 (70.0)	73 (45.3)	
Termino - terminal	107 (53.5)	16 (55.2)	3 (30.0)	88 (54.7)	
Colo anal manual anastomosis, n (%)	29 (14.5)	5 (17.2)	0	24 (14.9)	0.296
Intersphincteric resection (ISR), n (%)					
Total ISR	1 (0.5)	0	0	1 (0.6)	0.885
Partial ISR	8 (4)	1 (3.4)	0	7 (4.3)	
Diverting stoma, n (%)	179 (89.5)	25 (86.2)	10 (100.0)	144 (89.4)	0.735

*AL* Anastomotic leak, *SAL* symptomatic AL, *AAL* asymptomatic AL and *WAL* without AL, *TME* Total mesorectal excision; *PME* Partial mesorectal excision

### Short-term postoperative outcomes

**Fig. 1:**
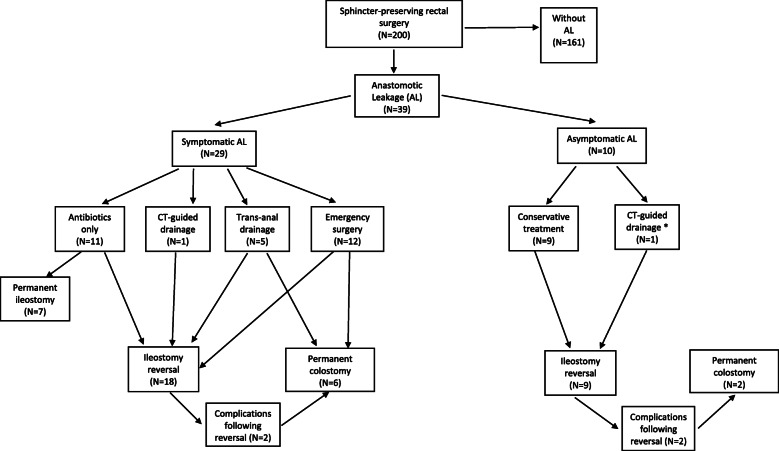
Short- and long-term postoperative outcome of all patients who underwent sphincter-preserving rectal cancer surgery. Symptomatic anastomosis leakage (SAL), acute management as well as asymptomatic anastomotic leakage (AAL) management is shown below. * One patient was diagnosed with AAL although presenting with pelvic collection on CT-scan and underwent CT-guided drainage

Postoperative AL occurred in 39 (19.5%) patients of whom 29 (14.5%) had SAL and 10 (5.0%) AAL. Stoma details in the different analysed groups are summarized in Fig. 1. Postoperative mortality rate in the total study population was nil. In the SAL group, sepsis was the main symptom in 25 (86.2%) patients. Additinonally, in four (13.8%) other SAL patient’s ileus (*n* = 2) and anal pus discharge (n = 2) were the only clinical symptoms observed. Symptomatic AL was diagnosed after a median of 10 (5.5–16.0) days postoperatively. Redo abdominal surgery was performed after a median of 7 (IQR 6.3–16.8) days in 12 (41.4%) patients (anastomosis resection and colostomy placement (*n* = 6) and peritoneal lavage with anastomosis repair and drainage (n = 6)). Three patients suffered from one or more organ dysfunctions after redo surgery and had therefore to be admitted to the intensive care unit. Trans-anal drainage was performed in 5 (17.2%) patients under general anesthesia. Overall, 11 (37.9%) patients were managed with antibiotics only. A permanent stoma was required in 13 (44.8%) patients (colostomy (n = 6), ileostomy (*n* = 7)).

The diagnosis of AAL was made by CT-scan after a median of 40 (IQR 69–194) days postoperatively. Three (30.0%) patients had stoma reversal before AAL was diagnosed (systematic CT-scan before reversal did not show signs of AL). Seven patients (70.0%) had AAL diagnosed before reversal. CT-scan drainage was required in one patient while the other 9 patients were treated conservatively with antibiotics only. Overall, nine patients underwent stoma reversal, of which 2 required permanent colostomy placement due to persistent fistula associated with unsatisfactory functional results. A total of three patients (30%) finally required a permanent stoma.

There were 18 (62.1%) patients who presented with severe postoperative complications in SAL group, with none in the AAL group and 9 (5.6%) patients without AL (*p* < 0.001). Severe postoperative complications in patients without AL occurred in 2 patients which had finally to undergo redo surgery for evisceration and small bowel ileus both on postoperative day seven.

A total of 161 (80.5%) patients were without AL (WAL group). Among WAL patients, 15 (9.3%) patients required permanent stoma placement (colostomy due to pelvic local tumor recurrence (*n* = 3), colostomy for perforation after dilatation of a anastomotic stenosis (*n* = 1) and ileostomy related to severe anastomosis stenosis (*n* = 11)). Postoperative details are summarized in Table 3 and in Fig. 1.

**Table 3:** Early postoperative outcome

N (%)	Overall Population	Symptomatic AL (SAL)	Asymptomatic AL (AAL)	Without AL (WAL)	P
	200 (100)	29 (14.5)	10 (5.0)	161 (80.5)	
Emergency surgery, n (%)	21 (11.5)	12 (41.4)	0	9 (5.6)	
Laparoscopic abdominal drainage, n (%)	3 (1.5)	3 (10.3)	0	0	
Open abdominal drainage, n (%)	5 (2.5)	3 (10.3)	0	2 (1.2)	0.001
Hartmann’s procedure, n (%)	6 (3.0)	6 (20.7)	0	0	
Ileostomy repair, n (%)	7 (3.5)	0	0	7 (4.3)	
Trans-anal drainage, n (%)	5 (2.5)	5 (17.2)	0	0	
CT-guided drainage, n (%)	2 (1.0)	1 (3.4)	1 (10,0)	0	0.003
Antibiotics treatment only, n (%)	23 (11.5)	11 (37.9)	9 (90.0)	0	–
Hospital stay (days) median ± IQR	11.0 (9.0–16.0)	19.0 (14.0–33.0)	11.5 (9.5–14.8)	10.0 (9.0–14.0)	< 0.001
Initial diverting stoma reversal, n (%)					
Yes	159 (88.8)	18 (72.0)	9 (90.0)	132 (91.7)	
No	20 (11.2)	7 (28.0)	1 (10.0)	12 (8.3)	0.021
Days before stoma reversal (days), median ± IQR	97.0 (74,1–188.2)	209.0 (127.3–327.3)	208.0 (115.7–274.7)	91.9 (69.9–138.4)	< 0.001

*AL* Anastomotic leak, *SAL* symptomatic AL, *AAL* asymptomatic AL and *WAL* without AL, *IQR* interquartile range

A primary diverting stoma reversal was perfomed in 132 (91.7%) patients without AL, 9 (90%) patients with AAL, and 18 (72%) patients with SAL, respectively (*p* = 0.021). The time intervall from primary surgery to stoma closure was similar in patients with SAL (209.0 (IQR 127.3–327.3)) and patients with AAL (208.0 (IQR 115.7–274.7)). However, this time span was significantly longer compared to patients without AL (91.9 (IQR 69.9–138.4) days, *p* < 0.001). Overall, complete and partial pathological responses were recorded in 28 (14.0%) and 113 (56.5%) patients with no differences observed between the three groups (*p* = 0.479). Resection margins were considered incomplete in 9 (4.5%) patients with again no differences between the three groups (*p* = 0.151). In patients without AL, three patients underwent colostomy placement after local pelvic tumor recurrence whereas one patient required colostomy due to a postinterventinal colonic perforation after dilatation of the stenotic anastomosis. Furthermore, another eleven other patients required an ileostomy for severe anastomotic stenosis.

### Long-term oncological outcomes

**Fig. 2:**
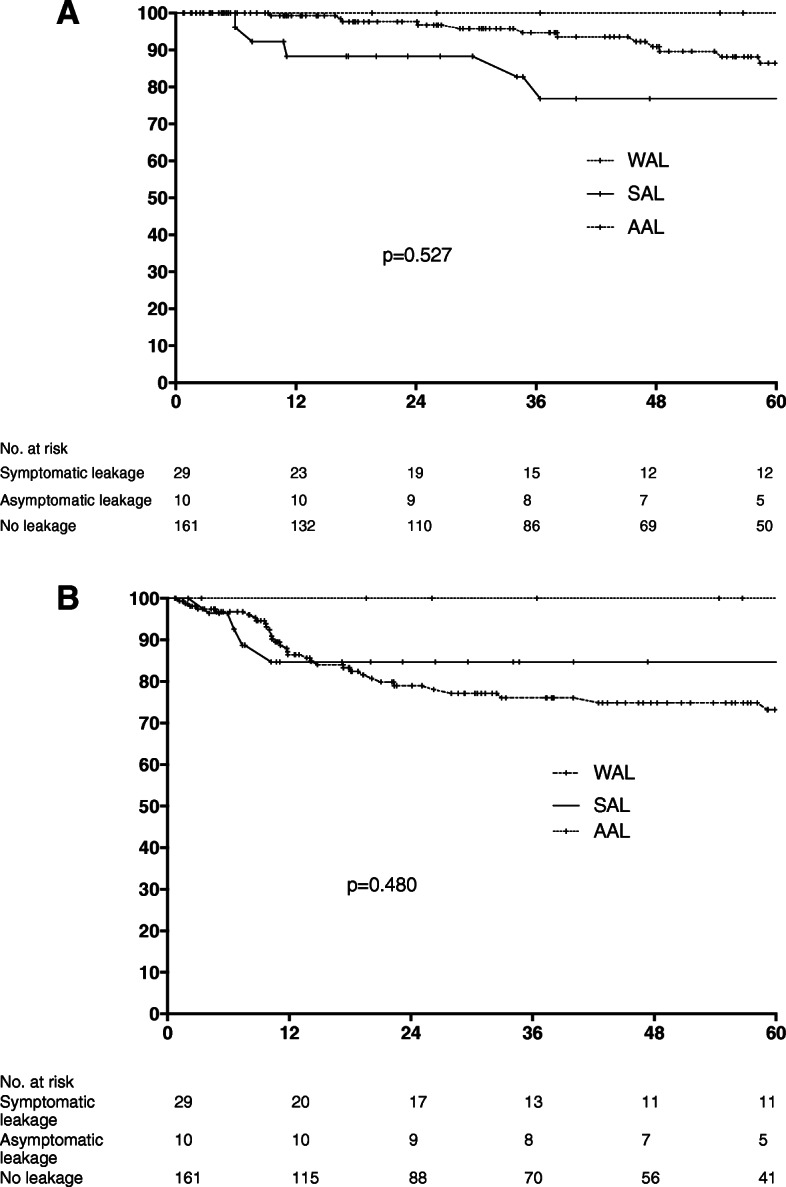
**a** Symptomatic anastomotic leak (SAL) group (solid line) and asymptomatic AL (AAL) group (Point line), without AL (WAL) group (Dash lines) Overall survivals in SAL, AAL and Without AL. Anastomotic leak (AL) groups symptomatic AL (SAL), asymptomatic AL (AAL) and without AL (WAL). X axis: months. Y axis: percentage survival. **b** Symptomatic anastomotic leak (SAL) group (solid line) and asymptomatic AL (AAL) group (Point line), without AL (WAL) group (Dash lines). Disease free survival in SAL, AAL and Without AL. Anastomotic leak (AL) groups symptomatic AL (SAL), asymptomatic AL (AAL) and without AL (WAL). X axis: month. Y axis: percentage survival

During the postoperative follow-up, after a median time interval of 10.7 months (IQR 8.8–18.8) after curative surgery, local and distal recurrences occurred in 10 (5.0%) and 34 (17.0%) patients, resepectively. No differences were found between the three groups (*p* = 0.170). Oncological results are detailed in Table 4. There were no patients in the AAL group who presented with local or distal recurrence. Disease-free survival comparison was therefore performed between the SAL and WAL group. No significant difference was observed. The 3-year disease-free survival rate was 86.6% for patients with SAL, 100% for those with AAL and 76% for those without AL (*p* = 0.480). At 5 years, the disease-free survival rate was similar in the three groups 84, 100 and 70%, respectively (p = 0.480). Overall, 52 (26.0%) patients underwent postoperative chemotherapy with no difference between the three groups (*p* = 0.465). Median follow-up was 38.0 months (0.7–151.5) and long-term mortality rate was 12.5% (*n* = 25) with no difference between the three groups (*p* = 0.743 and *p* = 0.543, respectively). Eight percent of patients died during follow-up from cancer progression (*n* = 16) and nine patients (4.5%) from other causes. The 3-year overall survival rate for patients in the SAL group was 82.9, 100% in the AAL group and 94.7% in the WAL group, resepectively (*p* = 0.527). Similarly, at 5-years, overall survival did not differ between the three groups (77, 100 and 88.3%; p = 0.527). Details about the overall and disease-free survival rates are presented in Fig. 2a-b.

**Table 4:** Pathology results

N (%)	Overall Population	Symptomatic AL (SAL)	Asymptomatic AL (AAL)	Without AL (WAL)	P
	200 (100)	29 (14.5)	10 (5.0)	161 (80.5)	
T stage, n (%)					
T0	28 (14.0)	2 (6.9)	2 (20.0)	24 (14.9)	0.460
T1	30 (15.0)	5 (17.2)	0	25 (15.5)	
T2	57 (28.5)	7 (24.1)	5 (50.0)	45 (27.9)	
T3	78 (39.0)	14 (48.3)	2 (20.0)	62 (38.5)	
T4	7 (3.5)	1 (2.9)	1 (10.0)	5 (3.1)	
N stage, n (%)					
N+	48 (24.0)	5 (17.2)	2 (20.0)	41 (25.5)	0.594
N0	152 (76.0)	24 (82.8)	8 (80.0)	120 (74.5)	
Pathological response, n (%)					
Absent	59 (28.5)	11 (38.0)	1 (10.0)	47 (29.2)	
Partial	113 (56.5)	17 (58.6)	7 (70.0)	89 (55.3)	0.479
Complete	28 (14.0)	1 (3.4)	2 (20.0)	25 (15.5)	
Lymph nodes, median ± IQR	18.0 (13.0–25.5)	18.0 (12.5–23.5)	18.5 (15.0–25.0)	17.5 (13.0–26.0)	0.593
Tumor diameter (mm), median ± IQR	25.5 (20.0–35.5)	27.5 (20.0–35.0)	30.0 (22.5–37.5)	25.0 (18.0–35.0)	0.971
Resection margins, n (%)					
Incomplete (R+)	9 (4.5)	3 (10.3)	1 (10.0)	5 (3.1)	
Complete (R0)	191 (95.5)	26 (89.7)	9 (90.0)	156 (96.9)	0.151

*AL* Anastomotic leak, *SAL* symptomatic AL, *AAL* asymptomatic AL and *WAL* without AL, *IQR* interquartile range

### Long-term functional results

**Fig. 3:**
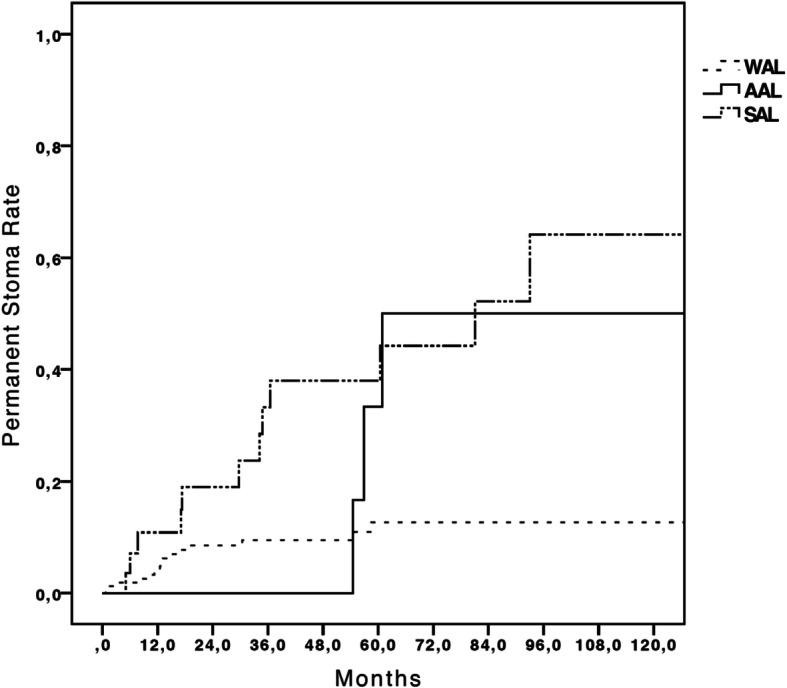
Permanent stoma rate determined as a time to event analysis in SAL, AAL and Without AL. Anastomotic leak (AL) groups symptomatic AL (SAL), asymptomatic AL (AAL) and without AL (WAL). X axis: month. Y axis: cumulative percentage of patients with permanent stoma

There were 31 (15.5%) patients requiring permanent stoma placement ((*n* = 15) WAL group, (*n* = 13) SAL group and (*n* = 3) in AAL group, *p* < 0.001)). In the WAL group, three patients underwent colostomy placement after local tumoral recurrence, one patient had colostomy for perforation after stenosis dilatation and eleven patients had ileostomies related to severe anastomosis stenosis. In patients with SAL, 6 patients had permanent stoma placement after postoperative redo surgery, 2 patients required colostomy placement after soma reversal for septic complication, and 5 patients required secondary colostomy placement after soma reversal for poor functional results. In patients with AAL, one patient required a permanent stoma due to medical comorbidities (cardiac and pulmonary insufficiency) and two other patients required a secondary colostomy placement after stoma reversal with poor functional results. Furthermore, analysis over time revealed a significantly different permament stoma rate between the three groups (*p* < 0.001) as shown in Fig. 3. Long-term functional results of 42 patients are shown in Table 5. Excluding deceased patients (*n* = 25) and patients with permanent stoma placement (*n* = 31), this represented a response rate of 28.2% for functional assessment scores. Only the Wexner continence grading scale was significantly higher in patients who presented with AL (*p* = 0.014). Wexner continence grading scale was also significantly different between the AAL, SAL and WAL group (*p* = 0.049). Permanent stoma rate was higher in patients with SAL compared to WAL patients (44.8% vs 9.3%, p < 0.001) and similar compared to the AAL group (30%, *p* = 0.699). Data are summarized in Fig. 3. Overall, patients with AL (*n* = 39) had a permanent stoma rate of 41% compared to 9.3% in the WAL group (*n* = 161), *p* < 0.001. Multivariate analysis showed that R1 resection and SAL were the only independent factors which were predictive for a permanent stoma (OR = 7.001 (1.067–45.930), *p* = 0.043 and OR = 8.209 (3.038–22.184), p < 0.001). Data are presented in Table 6.

**Table 5:** Late postoperative outcome

N (%)	Overall Population	Symptomatic AL (SAL)	Asymptomatic AL (AAL)	Without AL (WAL)	P
	200 (100)	29 (14.5)	10 (5.0)	161 (80.5)	
Oncological results					
Adjuvant chemotherapy, n (%)	52 (26.0)	7 (24.1)	1 (10.0)	44 (27.3)	0.465
Recurrence, n (%)					
Local	10 (5.0)	2 (6.9)	0	8 (5.0)	
Distance	34 (17.0)	3 (10.3)	0	31 (19.3)	0.170
Total patients with recurrence	39 (19.5)	4 (13.8)	0	35 (21.7)	
Time to recurrence (month), median ± IQR	10.7 (8.8–18.8)	7.0 (6.0–8.1)	0	11.8 (9.6–19.8)	0.173
Median of follow up (months) ± IQR	38.5 (17.2–65.6)	34.5 (17.1–66.5)	55.7 (28.8–61.9)	38.1 (16.7–65.5)	
Overall survival at 3 years	95.5%	82.9%	100%	94.7%	0.527
Overall survival at 5 years	91.5%	77%	100%	88.3%	
Disease free survival at 3 years	81.5%	86.6%	100%	76%	0.480
Disease free survival at 5 years	80.5%	84%	100%	70%	
Functional results					
Permanent stoma, n (%)	31 (15.5)	13 (44.8)	3 (30.0)	15 (9.3)	< 0.001
Ileostomy	18 (9.0)	7 (24.1)	1 (10.0)	11 (6.8)	
Colostomy	13 (6.5)	6 (20.7)	2 (20.0)	4 (2.5)	
Other late complications, n (%)					< 0.001
Stenosis	24	6	1	17	
Hernia	16	5	4	7	
Occlusion	3	1	0	2	
Mean of quality of life assessments					
LARS Score	26,2 ± 12,8	34,3 ± 4,0	28,8 ± 14,9	25,1 ± 13,0	0.522
Wexner Score	7,3 ± 4,8	10,3 ± 0,6	11,4 ± 3,8	6,4 ± 4,7	0.049
IIEF5	10,0 ± 8,9	18,0 ± 1,4	2,3 ± 2,3	10,4 ± 9,2	0.187
SF36	98,8 ± 10,5	105,0 ± 7,9	99,2 ± 5,4	98,2 ± 11,2	0.446
EORTC QLQ-C30	53,9 ± 11,4	49,3 ± 3,2	62,4 ± 14,5	53,0 ± 11,0	0.077
EuroQol	69,0 ± 18,9	75,0 ± 5,0	59,0 ± 18,2	69,9 ± 19,6	0.111
FIQL	86,8 ± 20,6	81,3 ± 3,5	74,2 ± 22,0	89,2 ± 20,8	0.136
EORTC QLQ-MY20	28,7 ± 7,0	28,3 ± 1,5	31,0 ± 7,1	28,4 ± 7,4	0.685

*AL* Anastomotic leak, *SAL* symptomatic AL, *AAL* asymptomatic AL and *WAL* without AL, *IQR* interquartile range)

**Table 6:** Multivariate analysis of factors associated with permanent stoma

	Odds Ratio	CI 95%	P
Age	1.041	0.989–1.097	0.127
ASA			
3	1.999	0.727–5.500	0.180
4	1.714	0.322–9.120	0.528
TME	2.586	0.875–7.643	0.086
R1	7.001	1.067–45.930	0.043
Fistula occurrence			
Asymptomatic fistula	3.221	0.718–14.456	0.127
Symptomatic fistula	8.209	3.038–22.184	< 0.001

## Discussion

In this retrospective single center study we conducted a comparative analysis of asymptomatic and symptomatic AL in patients who underwent rectal resection with curative intent. Both short-term and long-term morbidity, functional and oncological outcomes in this homogeneous population were analyzed. Patients suffering from SAL were found not to be at risk for an increased number of local and/or distant tumor recurrence rates compared to the group of AAL and WAL patients. However, in the group of patients suffering from postoperative anastomotic leakage (AAL and SAL) we observed an impaired long-term outcome of postoperative functional results. Furthermore, there is an increased risk for a permanent stoma in AAL and SAL patients which is as high as 41% and compated to patients with no postoperative anastomotic leakage (9.3%) is significantly higher.

The effect of postoperative anastomotic leakage (AL) on the oncological outcome following rectal cancer surgery is still debated controversially and remains unclear. Some authors have reported about reduced local recurrence rates whereas distant recurrence rates and overall survival rates were found to be similar. In contrast, equivalent local, distant disease recurrence rates and overall survival rates were observed by others [15, 35, 36]. Furthermore, two meta-analysis reported that AL was associated with higher local disease recurrence rates and reduced long-term cancer specific survival. No impact was found on the incidence of distant disease recurrence rates [15, 35]. Moreover, five pooled randomized trials [36] published in 2009 concluded that the disease-free survival was not affected by the postoperative appearance of symptomatic AL. However, symptomatic AL was observed to be associated with an impaired overall survival in such patients. Such discrepancies between the reported results in the aforementioned studies might be be explained by considering two important facts about postoperative AL in more detail.

First, in most previously published studies, any clinical impact of postoperative AAL was not assessed since no discrimination between AAL and SAL was made. So far, only a few studies analyzed the subgroup of AAL and observed that the short- as well as the and long-term outcome differed from the SAL and WAL group, respectively [16].

Second, the diagnosic work-up, definition of AAL and also the discrimination from patients without AL are still not clearly defined and are controversially discussed. Therefore it is obvious that the rate of patients with diagnosed postoperative AL varies considerably between the different studies with a reported prevalence which is somewhere in the range between 5% [37–39] to 28% [11–16].

In this present study, we observed an incidence of AL of 18% (39/200) which is indeed somewhat higher compared to other studies [37–39]. However, our findings could be explained by the fact that all patients in our study were subjected to AL screening which, in accordance with the recommendations of the working group of Panis et al., included routine blood testing and water soluble enema contrast CT-scan six weeks postoperatively and before stoma reversal [11–16].

In contrast to the study published by Hain et al. [16] we observed no significant difference of the overall and disease-free survival between the AAL, SAL and WAL group, respectively. Indeed, we are aware that an important limitation of our study, besides that it is retrospective, is the limited number of included patients which hampers statistical data interpretation. Nevertheless, the homogeneity of our study population is an important quality criterion compared to other studies which also included patients suffering from distant metastatic disease [16, 38, 39]. Including such patients suffering from distant metastases carries a relevant risk to bias study data since such patients suffer from impaired postoperative anastomotic healing with all the corresponding known negative sequelae [14]. Finally, the management of rectal cancer has substantially improved in the last decade (i.e. rectal surgery, neoadjuvant treatment, diagnosis and treatment of AL). Therefore, data from previous studies (1971 to 2010) [37–39] are not comparable to present data since they do not reflect current standards for diagnosis and treatment of rectal cancer. Hence, the interpretation of our current data in the context of such previous studies is probably of limited relevance.

Although we did not find a negative impact of SAL and AAL on the oncological outcome in our present study we observed an impaired short- and long-term functional outcome of AAL compared to patients without AL (WAL). This is again in contrast to a recently published study by Hain et al. which found no difference between the LARS score in the AAL, SAL, and WAL group, respectively. However, the authors of this study did not discriminate between WAL and AAL in their multivariate analysis of predictive factors of the postoperative functional outcome [11]. Although it is obvious that AL has a negative impact on the postoperative functional outcome [40–45] it remains still unclear for the subgroup of patients with AAL. Similar findings as ours were found by Lim et al. who reported that the bowel function after ileostomy closure was equally impaired in the AAL and SAL group, respectively [40].

In our study, the postoperative time interval to stoma reversal was similar in SSL and AAL, but significantly longer compared to WAL patients. These results are consistent with findings in other previous studies [11, 40]. Furthermore, we observed that the permanent stoma rates were quite similar in the AAL (30%) and SAL (44.8%) group but significantly higher compared to the WAL group (9.3%). Some other authors have reported that all AAL patients can be managed conservatively with spontaneous healing whereas SAL patients showed only a 40% percent rate of spontaneous healing [40]. An important finding in our study is the fact that, even if AAL patients can be managed conservatively, the functional postoperative outcome can be impaired.

Our multivariate analysis revealed that SAL and R1 rectal tumor resection were the only independent risk factors for a permanent stoma. For the presence of AAL only a trend towards a higher stoma rate was found.

We are aware about some limitations of our study (small number, retrospective). Nonetheless, our study is the first which observed that AAL patients have a risk for worse postoperative functional long-term outcome and a high risk for a permanent stoma. Finally, previous studies are lacking of long-term outcome data for AAL and SAL and are heterogeneous since they also included patients suffering from distant metastatic disease [11–18].

## Conclusion

In conclusion, AL did not impair long-term oncological results (disease-free and overall survival) in patients with rectal adenocarcinoma. Despite the small numbers of patients, especially in the AAL group, long-term functional results were impaired by the occurrence of SAL and AAL with a similar permanent stoma rate in both groups.

## Data Availability

The datasets used and analyzed during the current study are available from the corresponding author on reasonable request.

## Abbreviations

TME: Total Mesorectal Excision
ASA: American Society of Anesthesiology score
EUS: Endorectal Ultrasound
MRI: Magnetic resonance imaging
CT: Cumputed tomography
RCT: Radio chemo therapy
AL: Anastomotic Leakage
SAL: Symptomatic AL
AAL: Asymptomatic AL
WAL: Without AL
